# Scutellarin Improves Type 2 Diabetic Cardiomyopathy by Regulating Cardiomyocyte Autophagy and Apoptosis

**DOI:** 10.1155/2022/3058354

**Published:** 2022-05-06

**Authors:** Yanmei Su, Xiaoming Fan, Siman Li, Zhigang Li, Ming Tian, Shude Li

**Affiliations:** ^1^Department of Cell Biology and Medical Genetics, School of Basic Medicine, Kunming Medical University, Kunming, Yunnan 650500, China; ^2^Kunming Angel Women's and Children's Hospital, Kunming, Yunnan 650031, China; ^3^Gaungxi Key Laboratory of Diabetic Systems Medicine, Guilin Medical University, Guangxi Zhuang Autonomous Region 541004, China; ^4^Department of Human Anatomy, School of Basic Medicine, Guilin Medical University, Guangxi Zhuang Autonomous Region 541004, China; ^5^Department of Biochemistry and Molecular Biology, School of Basic Medicine, Kunming Medical University, Kunming, Yunnan 650500, China

## Abstract

Diabetes cardiomyopathy has metabolic disorder and abnormality of cardiomyocytes, which is closely related to autophagy or apoptosis of cardiomyocytes. Scutellarin (SCU) is an important monomer extracted from *Erigeron breviscapus* (vant.) Hand.-Mazz. This study was conducted to investigate the function of SCU on apoptosis and autophagy of myocardial cells. We established a model of type 2 diabetic cardiomyopathy by high-fat and high-sugar diet. The results indicated that SCU downregulated blood glucose, total cholesterol (TC), triglyceride (TG), and low-density lipoprotein (LDL) levels and upregulated high-density lipoprotein (HDL) level. In addition, SCU downregulated lactic dehydrogenase 1 (LDH1) and creatine kinase (CK) levels. Meanwhile, SCU improved the myocardium morphology and reduced myocardial apoptosis. Furthermore, SCU promoted the mRNA and protein expression of autophagy-related factors (Beclin-1 and LC3-II) and inhibited the mRNA and protein expression of apoptosis-related factors (caspase-3, caspase-8, caspase-9, caspase-12, Bax, and Cyt-C). In conclusion, SCU can promote autophagy signal pathway by upregulating the autophagy-related factors and inhibit the apoptotic signal pathway by downregulating apoptosis-related factors, thereby relieving type 2 diabetic cardiomyopathy (T2DC).

## 1. Introduction

Type 2 diabetic cardiomyopathy (T2DC) is one of the causes of death in patients with type 2 diabetes mellitus [1]. Among them, diabetes cardiomyopathy is a long-term concurrent disease [2]. Therefore, this disease has become one of the diseases that researchers are more interested to study in recent years. T2DC has metabolic disorder and abnormality of cardiomyocytes, which is closely related to autophagy or apoptosis of cardiomyocytes [3, 4]. Autophagy and apoptosis play important roles in the development of various cardiovascular diseases [5, 6]. Autophagy can inhibit apoptosis by degrading the activation of apoptosis-related proteins. In turn, the activation of apoptosis-related proteins can suppress autophagy-related proteins (such as LC3-II and Beclin-1) [7, 8]. At present, the complex counter-regulation mechanisms that mediate autophagy and apoptosis are not fully understood. Therefore, it has become an important research direction to look for the relationship between the apoptosis and autophagy and to explore drugs to improve type 2 diabetes complicated with myocardial injury.

Scutellarin (SCU) is an important monomer from *Erigeron breviscapus* (vant.) Hand.-Mazz. Studies have reported that SCU inhibited cisplatin-induced apoptosis by decreasing the activation of caspase-3, p53, and the ratio of Bax/Bcl-2. In addition, SCU can prevent cisplatin-induced suppression of autophagy by enhancing LC3-II/LC3-I and Atg7 and inhibiting Beclin-1 [9]. Besides, SCU can improve diabetic nephropathy by reducing ROS production and reducing myocardial apoptosis by upgrading the expression of antiapoptotic protein Bcl-2 [10, 11]. However, it has not been reported whether SCU can inhibit apoptosis by promoting cardiomyocyte autophagy and improving the T2DC.

In this study, the T2DC model was established to explore the relationship between autophagy and apoptosis in the process of myocardial cell damage, and the effect of SCU on improving myocardial injury, which provides experimental evidence for the treatment of diabetic myocardial injury by SCU.

## 2. Material and Method

### 2.1. Reagents, Antibodies, and Animal

Scutellarin (purity 98%) was purchased from Honghe Qianshan Bioengineering Co., Ltd. (Honghe, China). Streptozocin (STZ) and rosiglitazone were obtained from Multi Sciences (Lianke) Biotech, Co., Ltd. (Hangzhou, China). Antibodies to detect *β*-actin, procaspase-3, procaspase-8, procaspase-9, cleaved caspase-3, cleaved caspase-8, cleaved caspase-9, Bax, Cyt-C, Bcl-2, Beclin-1, and LC3-II were ordered from Santa Cruz Biotechnology. The procaspase-12 polyclonal antibody was ordered from Abcam Company. Adult male Sprague-Dawley (SD) rats (180 to 200 g) were obtained from the Experimental Animal Center of Kunming Medical University, China. Two weeks after the adaptive feeding of the rats, they were randomly divided into control group, model group, SCU low-dose (100 mg/kg/d), high-dose (200 mg/kg/d) treatment group, and rosiglitazone (5 mg/kg/d) positive control group (each group of ten). The model group was given a high-sugar and high-fat diet for 8 weeks and injected with STZ by 35 mg/kg intraperitoneally, while the control group was given a normal diet. The SCU groups were administered orally for 8 weeks [12].

### 2.2. Biochemical Index Detection and Morphological Staining

The level of glucose in the serum was detected by a glucose meter (Accu-Check Performa, Germany). The levels of lactic dehydrogenase 1 (LDH1), creatine kinase (CK), total cholesterol (TC), triglyceride (TG), low-density lipoprotein (LDL), and high-density lipoprotein (HDL) in the serum were measured by an automatic biochemical analyzer. The change of morphology was detected by hematoxylin-eosin staining (HE), immunohistochemical (IHC), and terminal-deoxynucleoitidyl transferase-mediated nick end labeling (TUNEL) staining.

Taken a small piece of tissue and fixed it in 10% paraformaldehyde. After washing with tap water, it was subjected to a series of operations such as dehydration, transparency, paraffin embedding, and sectioning for HE staining. For immunohistochemical detection, paraffin sections were repaired with citric acid, and the antibody was added to each section, respectively (LC3-II, 1 : 100; Beclin 1, 1 : 100) at 4°C incubation overnight. Secondary antibody was added and incubated at room temperature for 15 min. After staining with hematoxylin for 10 min, it was differentiated with ethanolic hydrochloric acid for 20 s and rinsed with water for 10 min. After dehydration with ethanol, it was made transparent with xylene, sealed with a neutral gel. For TUNEL staining, after dewaxing the liver paraffin section, the section was added to the proteinase K for 30 min, and the mixture solution of TdT enzyme and biotin-dUTP (TdT : biotin − dUTP = 1 : 9) was incubated in 37°C for 1 h, and then, the converter-AP solution was added in 37°C incubated for 20 min. The 5-bromo-4-chloro-3-indolyl phosphate/nitroblue tetrazolium chloride (BCIP/NBT) solution and the nuclear fast red were subjected to a color reaction, and the AEC aqueous sealing tablets were used for sealing [12].

### 2.3. Real-Time Quantitative PCR

Total RNA was extracted from the heart tissues using the Total RNA Extractor (TRIzol) kit. cDNA of each RNA sample was reverse-transcribed with the M-MLV RTase cDNA kit according to the manufacturer's instruction. Real-time quantitative PCR (RT-qPCR) was performed using SYBR Premix EX Tap 2x kit in CFX 96 RT-qPCR system. The reaction conditions were as follows: 95°C for 30 s, 95°C for 5 s, and 60°C for 30 s, followed by 30 cycles and then 72°C for 10 min according to our previous research methods [13, 14]. Primer sequences are shown in Table 1.

### 2.4. Western Blotting

Total protein was extracted with RIPA lysate added with PMSF and then quantified by BCA, and finally, the protein concentration was pulled to 5 *μ*g/*μ*L. The protein was loaded at 40 *μ*g, and the protein was separated by SDS-PAGE 10% gel and transferred to a polyvinylidene fluoride (PVDF) membrane and blocked with 5% milk for 120 min at room temperature. Then, the membrane was incubated with cleaved caspase-3, cleaved caspase-8, cleaved caspase-9, cleaved caspase-12, procaspase-3, procaspase-8, procaspase-9, procaspase-12, Bax, Cyt-C, Bcl-2, Beclin-1, and LC3-II antibody overnight at 4°C. All of them diluted by a ratio of 1 : 1000. The secondary antibody was horseradish peroxidase- (HRP-) conjugated anti-rabbit or mouse IgG (1 : 5000 dilution). The membrane was visualized by enhanced chemiluminescence (ECL) detection system. The signal intensity was quantified using ImageJ software.

### 2.5. Statistical Analysis

All data were analyzed using SPSS 13.0 software. Measurement data were expressed as the mean ± standard deviation. The data were measured by the mean ± standard error of the mean (SEM) of the data. One-way ANOVA and Tukey's post hoc test were used for comparison among multiple groups. *P* < 0.05 indicates that the difference was statistically significant.

## 3. Results

### 3.1. SCU Inhibits the Damages of Cardiomyocytes in T2DC Model

**Figure 1:**
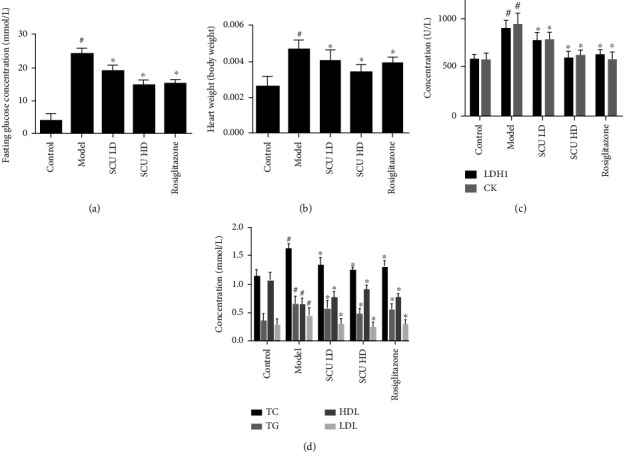
SCU reduces blood glucose and lipid factors and improves cardiac function in the T2DC model. (a) SCU reduced the concentration of fasting glucose in the serum. (b) SCU reduced heart weight/body weight. (c) SCU reduced the concentration of LDH1 and CK. (d) SCU suppressed lipid accumulation. The values shown are the mean ± standard error of the mean (SEM) of the data from three independent experiments. ^#^Significant compared with the control group alone, *P* < 0.05. ^∗^Significant compared with the model group alone, *P* < 0.05.

**Figure 2:**
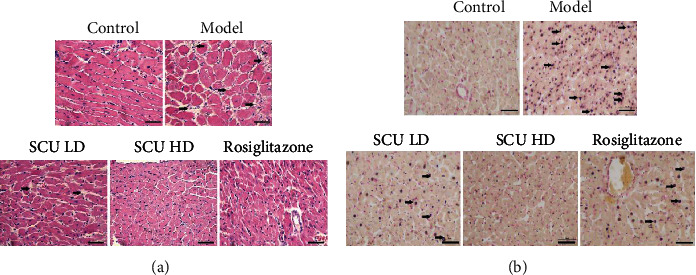
SCU reversed cardiac pathological changes and inhibited cardiomyocyte apoptosis in the T2DC model. (a) SCU reversed the pathological changes of cardiomyocytes by HE staining assay. (b) SCU inhibited the apoptosis of cardiomyocytes by TUNEL staining assay.

Firstly, we examined the effects of SCU in the T2DC model. An antidiabetes drug, rosiglitazone, was used as the control agent. The results indicated that SCU reduced the fasting glucose and the ratio of the heart weight/body weight, inhibited LDH1 and CK levels, and improved cardiac function in a dose-dependent manner (Figures 1(a), 1(b), and 1(c)). SCU also reduced TG, TC, and LDL levels and raised HDL levels, alleviating the accumulation of lipids in a dose-dependent manner (Figure 1(d)). In addition, SCU gradually normalizes the cardiomyocyte arrangement of the T2DC model in a dose-dependent manner by HE staining assay (Figure 2(a)). Meanwhile, SCU inhibits cardiomyocyte apoptosis in a dose-dependent manner by TUNEL staining assay (Figure 2(b)). The results indicated that SCU alleviated myocardial damage in the T2DC model.

### 3.2. SCU Suppresses the Apoptotic Signaling Pathway in the T2DC Model

**Figure 3:**
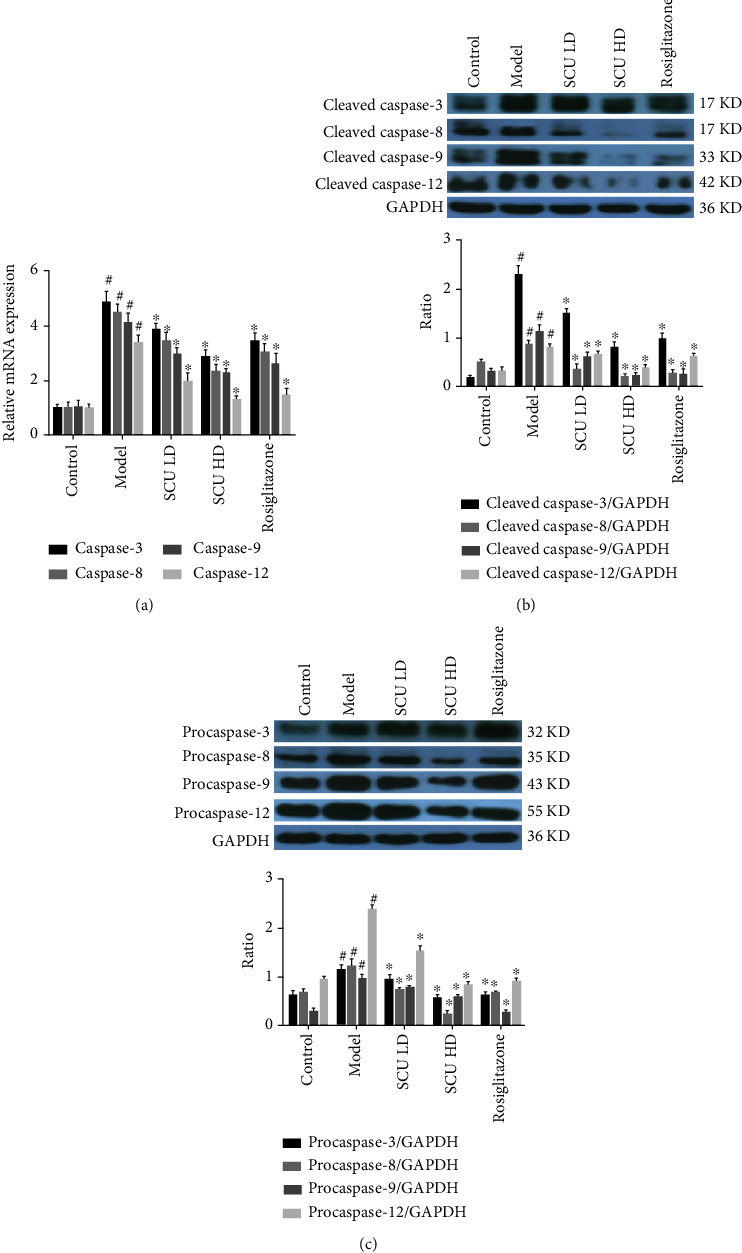
SCU suppresses the apoptotic signaling pathway in the T2DC model. (a) SCU suppressed the mRNA expression of caspase-3, caspase-8, caspase-9, and caspase-12 by RT-qPCR assay. (b) SCU suppressed the activity of caspase-3, caspase-8, caspase-9, and caspase-12 by western blot detection. (c) SCU inhibited the protein expression of procaspase-3, procaspase-8, procaspase-9, and procaspase-12 by western blot assay. The values shown are the mean ± standard error of the mean (SEM) of the data from three independent experiments. ^#^Significant compared with the control group alone, *P* < 0.05. ^∗^Significant compared with the model group alone, *P* < 0.05.

**Figure 4:**
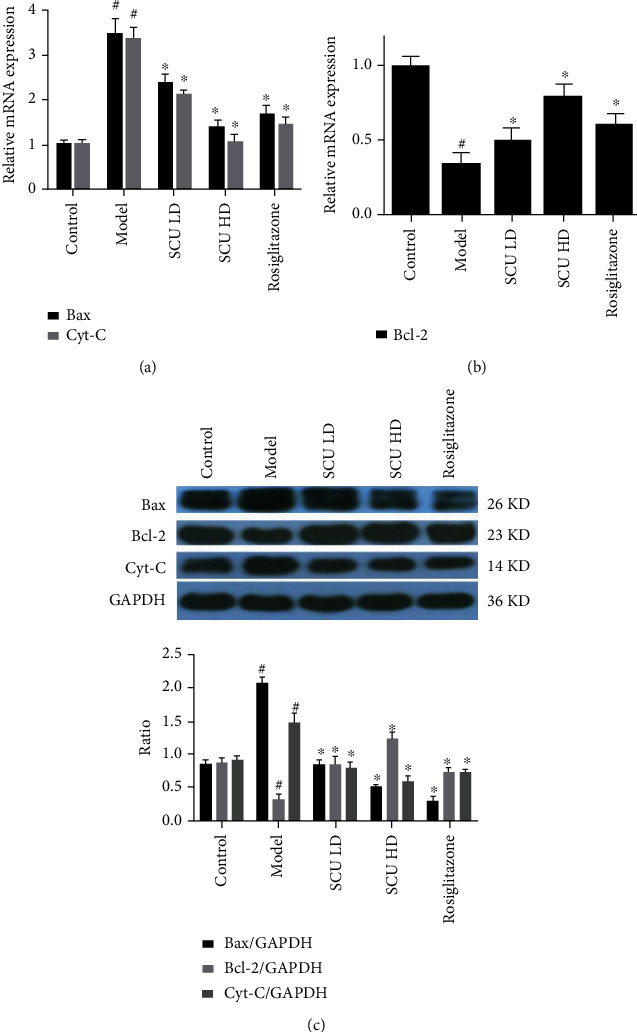
SCU suppresses the expression of Bax and Cyt-C and promotes the expression of Bcl-2. (a) SCU suppressed the mRNA expression of Cyt-C and Bax by RT-qPCR assay. (b) SCU promoted the expression of Bcl-2 mRNA by RT-qPCR assay. (c) SCU inhibited the expression of Cyt-C and Bax protein and promoted the expression of Bcl-2 protein by western blot test. The values shown are the mean ± standard error of the mean (SEM) of the data from three independent experiments. ^#^Significant compared with the control group alone, *P* < 0.05. ^∗^Significant compared with the model group alone, *P* < 0.05.

Furthermore, we detected the factors associated with apoptosis. Firstly, we detected the mRNA expression of caspase-3, caspase-8, caspase-9, and caspase-12. The results showed that SCU inhibited the mRNA expression of caspase-3, caspase-8, caspase-9, and caspase-12 in a dose-dependent manner (Figure 3(a)). Next, we examined the effect of SCU on the activity of studied caspase families by western blot. The results demonstrated that SCU suppressed the activity and protein expression of caspase-3, caspase-8, caspase-9, and caspase-12 in a dose-dependent manner (Figures 3(b) and 3(c)). In addition, we further detected other apoptosis factors (Bax, Bcl-2, and Cyt-C) by RT-qPCR and western blot. The results indicated that SCU suppressed the levels of mRNA and protein expression of Bax and Cyt-C, meanwhile increased the levels of mRNA and protein of Bcl-2 in a dose-dependent manner (Figure 4). The above results demonstrated that SCU suppressed myocardial cell apoptosis in the T2DC model.

### 3.3. SCU Promotes the Autophagy Signal Pathway in the T2DC Model

**Figure 5:**
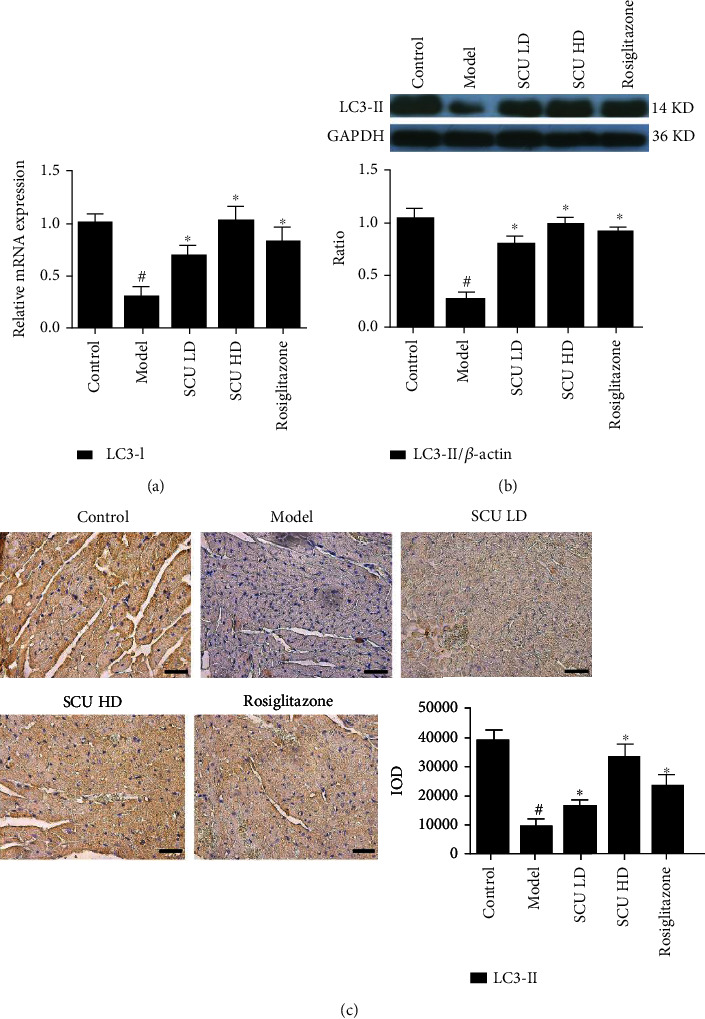
SCU upregulates the expression of the autophagy factor LC3-II. (a) SCU upregulated the mRNA expression of LC3-I by RT-qPCR assay. (b) SCU upregulated the protein of LC3-II by western blot detection. (c) SCU upregulated the protein of LC3-II by HIC assay. The values shown are the mean ± standard error of the mean (SEM) of the data from three independent experiments. ^#^Significant compared with the control group alone, *P* < 0.05. ^∗^Significant compared with the model group alone, *P* < 0.05.

**Figure 6:**
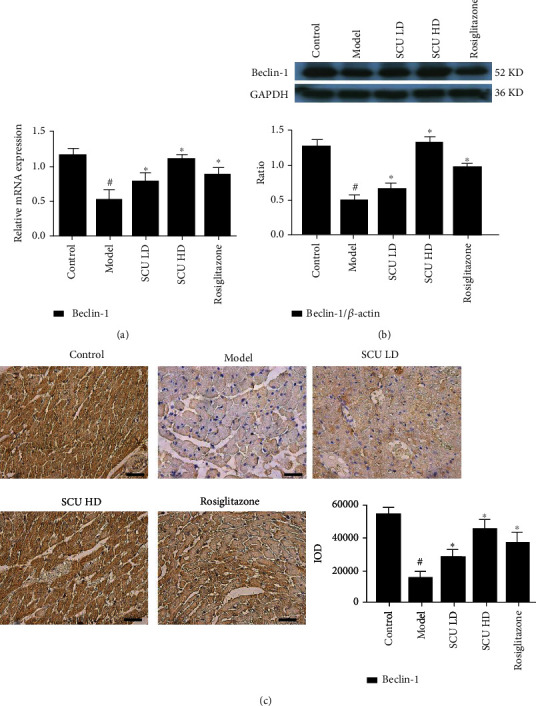
SCU upregulates the expression of the autophagy factor Beclin 1. (a) SCU upregulated the mRNA expression of Beclin 1 by RT-qPCR assay. (b) SCU upregulated the protein expression of Beclin 1 by western blot assay. (c) SCU upregulated the protein expression of Beclin 1 by HIC assay. The values shown are the mean ± standard error of the mean (SEM) of the data from three independent experiments. ^#^Significant compared with the control group alone, *P* < 0.05. ^∗^Significant compared with the model group alone, *P* < 0.05.

Since apoptosis and autophagy are closely related, we further examined the effect of SCU on cardiac autophagy in the diabetic cardiomyopathy model. RT-qPCR results showed that SCU increased the mRNA expression of the autophagy-related factors LC3-I and Beclin-1 in a dose-dependent manner (Figure 5(a)). Furthermore, western blot and IHC results showed that SCU also increased the levels of LC3-II protein in a dose-dependent manner (Figures 5(b) and 5(c)). We found that SCU has a similar effect on another autophagy-related factor Beclin-1 (Figure 6). The above results showed that SCU promoted the autophagy of myocardial cells in the diabetic cardiomyopathy model.

## 4. Discussion

Tissue and organ function damage is the main complication in the occurrence and development of diabetes, among which diabetic cardiomyopathy is one of the serious complications [15]. The caspase family is an important factor in carrying out apoptosis [16]. Caspase-3 is a key factor in apoptosis [17]. Caspase-8 is mainly involved in apoptosis induced by exogenous death receptor pathways [18]. Bcl-2/Bax and Cyt C are mainly involved in mitochondria-mediated apoptosis [19], while caspase-9 and caspase-12 take part in endoplasmic reticulum stress-associated apoptosis [20]. Previous studies have shown that MGE IIe regulates the activity of these apoptotic factors in the T2DC model, but the relationship between apoptosis and autophagy has not been studied in this model [21].

Autophagy activates to maintain cell survival after multiple stimuli (a nutrient deficiency, hypoxia, peroxidation, viral infection, etc.) [22]. When the stimulation is persistent existence or fatal, the autophagy is difficult to relieve the intracellular stress levels or autophagosome function is impaired, and apoptosis or nonprogrammed death pathways are activated [23]. Autophagy was inhibited after apoptosis was initiated. Apoptosis protein of the caspase cleaves ATG protein and Beclin-1, making it loses the ability to induce the autophagy and inhibit the autophagy level. Then, the autophagy protein fragments obtained the proapoptotic function [24, 25]. *Bcl-2* is an antiapoptotic gene that can be combined with the autophagy gene *Beclin-1* to enhance the activity of the autophagy factors such as the protein of LC3-I turned into LC3-II and thus inhibit the generation of cell apoptosis [26, 27]. Crosstalk between the autophagy and apoptosis is complex and critical to cell fate. Although the complex reverse regulation mechanisms that mediate the apoptosis and autophagy are not fully understood, the important points of crosstalk include the interaction between Beclin-1 and Bcl-2, the interaction mediated by the caspase and Ca^2+^ protease, the cleavage of the autophagy-related proteins, and the autophagy degradation of the caspases. According to the above correlation between autophagy and apoptosis, it has become an important direction of research to explore the effects of autophagy and apoptosis on myocardial injury in T2DC, to search for natural drugs with low toxicity and therapeutic effects on T2DC.

In the process of diabetes research, the establishment of animal models is an important basis for research [28]. We established the T2DC model through this method and used the drug SCU treatment of cardiovascular and cerebrovascular diseases as the object of study to explore whether SCU can alleviate diabetic cardiomyopathy as well as the underlying mechanisms involved in the autophagy and apoptotic molecules.

The results showed that SCU decreased gradually the ratio of the heart weight and body weight and downregulated the blood glucose, TC, TG, LDL, LDH1, and CK levels and upregulated HDL levels in a dose-dependent manner. HE staining results indicated that SCU improved the disorder of cardiac cells and decreased the interspace of cardiomyocytes. Furthermore, the signaling pathways of the apoptotic and autophagy results indicated that the levels of protein and mRNA of caspase-3, caspase-8, caspase-9, caspase-12, Bax, and Cyt-C in myocardial cells gradually decreased, while the expression of the cleaved protein of caspase-3, caspase-8, caspase-9, and caspase-12 also decreased by the treatment of SCU. Moreover, the mRNA or protein expression of Bcl-2, Beclin-1, LC3-I, and LC3-II increased after being treated with SCU.

In conclusion, SCU may alleviate diabetic cardiomyopathy by upregulating the autophagy-related factors (Beclin-1, LC3-I, and LC3-II) and downregulating apoptosis-related factors (caspase-3, caspase-8, caspase-9, caspase-12, Bax, and Cyt-C) of cardiomyocytes. However, there are some limitations to this study, as the experimental study focused on the apoptosis and autophagy pathway of cardiomyocytes, whereas other pathways are also involved in T2DC. Moreover, this study only conducted experiments using an animal model and did not perform related cell experiments. In subsequent research, we intend to further explore the pharmacological mechanism of SCU in the treatment of T2DC *in vitro* and *in vivo*.

## Figures and Tables

**Table 1:** Primer sequences required for experiments.

Name	Sequence	Tm	Amplicon length
*Procaspase-3*	F: GGATTACCCTGAAATGGGCTTGT	60°C	157
	R: CTCTGAGGTTAGCTGCATCGACAT		
*Procaspase-8*	F: AGGCACAGCACCGCTTT	60°C	123
	R: GTCTACGGAACGGAGGG		
*Procaspase-9*	F: TTCCTCGCTTCATCTCCTGCTTA	60°C	178
	R: TTGATTTGAGTCCCATTGGTCCC		
*Procaspase-12*	F: TTGGATACTCAGTGGTGATAAAGGA	60°C	146
	R: GGATGCCGTGGGACATAAAGA		
*Bcl-2*	F: ACTTCTCTCGTCGCTACCGTCG	60°C	181
	R: CCCTGAAGAGTTCCTCCACCACC		
*Bax*	F: CATGAAGACAGGGGCCTTTTTG	60°C	170
	R: TCAGCTTCTTGGTGGATGCGTC		
*LC3-I*	F: GCGCCGGATGATCTTGAC	60°C	153
	R: CTTCGCCGACCGCTGTAA		
*Beclin-1*	F: AATCTTGCCTTTCTCCAC	60°C	145
	R: TTGCCGTTGTACTGTTCT		
*Cyt-C*	F: GCGCCGGATGATCTTGAC	60°C	163
	R: CTTCGCCGACCGCTGTAA		
*GAPDH*	F: TCTCTGCTCCTCCCTGTTC	60°C	161

## Data Availability

The underlying data supporting the results of our study can be found in our original data.
